# Burnout Syndrome and shift work among the nursing staff^1^

**DOI:** 10.1590/1518-8345.2550.3022

**Published:** 2018-08-09

**Authors:** Viviane Vidotti, Renata Perfeito Ribeiro, Maria José Quina Galdino, Julia Trevisan Martins

**Affiliations:** 2 MSc, RN, Hospital Evangélico de Londrina, Londrina, PR, Brazil.; 3 PhD, Adjunct Professor, Departamento de Enfermagem, Universidade Estadual de Londrina, Londrina, PR, Brazil.; 4 Doctoral student, Universidade Estadual de Maringá, Maringá, PR, Brazil. Assistant Professor, Departamento de Enfermagem, Universidade Estadual do Norte do Paraná, Bandeirantes, PR, Brazil.; Universidade Estadual do Norte do Paraná, Departamento de Enfermagem, Universidade Estadual do Norte do Paraná, Bandeirantes, PR, Brazil; 5 PhD, Associate Professor, Departamento de Enfermagem, Universidade Estadual de Londrina, Londrina, PR, Brazil.

**Keywords:** Nursing, Shift Work, Workplace, Burnout, Professional, Stress Psychological, Occupational Health

## Abstract

**Objective:**

to analyze the factors associated with Burnout Syndrome among nursing workers according to work shift.

**Method:**

cross-sectional study addressing a representative sample of 502 nursing workers from a philanthropic hospital facility. Data were collected using a characterization instrument, the Maslach Burnout Inventory - Human Service Survey and the Demand-Control-Support Questionnaire. Data were analyzed using descriptive statistics and multiple binary logistic regression.

**Results:**

levels of Burnout Syndrome were significantly higher among those working the day shift and associated factors included: high demand; low control; low social support; dissatisfaction with sleep and financial resources; being a nurse; and sedentariness. Professionals working the night shift, having low social support, being dissatisfied with sleep, having children, not having a religion, having worked for a short period in the institution, and being a nursing technician or aid were significantly more likely to experience high levels of the syndrome*.*

**Conclusion:**

psychosocial factors and factors from the work context, mainly low social support, were associated with the syndrome dimensions among nursing workers of both shifts.

## Introduction

Shift work is necessary and indispensable in hospitals to ensure the continuity of care delivered to patients. In this sense, nursing workers are among the professionals who need to conform to this form of labor organization because nurses are required to provide care 24 hours every weekday^1^. Shift work, however, has been associated with changes in biological functions that lead to physical and mental disorders^2^ ^-^ ^3^. 

In addition to shift work, nursing workers also experience a fragmented work process, interpersonal relationships that are often conflictive, low wages, a highly demanding environment, insufficient human and technological resources, emotional stress, and they witness suffering and death daily. Thus, they are often faced with factors that generate occupational stress with the potential to affect their mental health^4^ ^-^ ^5^. 

Therefore, Burnout Syndrome is a psychosocial phenomenon emerging among nursing workers in response to their complex work environments^6^. This syndrome is composed of three dimensions: emotional exhaustion, understood as a lack of energy and a sense that emotional resources have been exhausted; depersonalization, characterized by emotional detachment; and low professional realization, a tendency of individuals to self-assess their work performance negatively and a dissatisfaction with their careers^7^. Hence, it is characterized by a loss of meaning at work, lack of motivation, negative attitudes, and detachment from others, which harm the work process in the health field^8^ ^-^ ^9^. 

Despite the prevalence of Burnout Syndrome and the fact that nursing work in hospitals is performed as shift work, Brazilian studies investigating this relationship have only addressed nurses and specific sectors, with divergent results^10^ ^-^ ^11^. International studies only report that work shifts of more than 12 hours increase the levels of Burnout Syndrome^12^ ^-^ ^13^. Thus, it is important to broaden the settings where studies take place to address the entire nursing staff and verify factors associated with the syndrome according to the day and night shifts^3^ ^,^ ^14^. These studies are important to supporting managers and nursing workers in order to devise measures intended to improve the working conditions for the different work shifts and enable greater quality of life at work.

In accordance with the preceding discussion, this study’s objective was to analyze the factors associated with Burnout Syndrome among nursing workers according to work shift.

## Method

Cross-sectional study conducted in a general hospital in a city in the state of Paraná, Brazil. This philanthropic hospital provides care of medium and high complexity and has 347 beds distributed among the hospitalization ward, intensive care unit, intermediate care unit, maternity ward, pediatrics, emergency room, and surgical center. 

The study population was composed of 698 nursing professionals working in this facility. Inclusion criteria were providing direct care to patients and having worked in this facility for at least one year. Exclusion criteria were working exclusively in head positions or being on leave.

Based on this number of workers, the sample size was calculated adopting a 95% confidence interval and maximum error of 5%, which resulted in a minimum of 219 workers. A total of 510 workers met the inclusion criteria, 8(1.57%) of whom refused to participate. Of the 502 participants remaining, 193(38.44%) were nurses, 273(54.38%) were nursing technicians and 36(7.18%) were nursing aids; 271(53.98%) worked on the day shift (7am-13pm or from 13pm-19pm), while 231(46.02%) worked on the night shift (7pm-7am). Note that 86 workers had a second job, but the work shift of this second job was the same as that in which they worked in this facility; that is, all those working on the day shift, exclusively worked in the day shift in all their jobs. The same was true for those working on the night shift.

From August to November 2016, these workers were invited to a private room on the facility’s premises to receive clarification about the study. Those who consented and signed free and informed consent forms received an envelop containing an instrument intended to characterize the participants, the Maslach Burnout Inventory - Human Service Survey (MBI-HSS) and the Demand-Control-Support Questionnaire (DCSQ). After completing the instruments, they were asked to drop the envelop inside an urn located in the same room to ensure confidentiality.

A questionnaire was developed to address sociodemographic and occupational characterization, identify life habits, age, sex, marital status, children, religion, education, work shift, profession, weekly workload, years working in the facility, number of jobs, monthly income, physical activity (frequency and duration), smoking, satisfaction with sleep patterns, leisure and financial resources. 

The MBI-HSS is a self-reported questionnaire with 22 items assessing Burnout Syndrome by considering three dimensions: emotional exhaustion (nine items), depersonalization (five items) and personal fulfillment (eight items). The frequency with which the respondents experience certain situations in their workplaces was determined on a six-point Likert scale. Predisposition to Burnout Syndrome is seen as a combination of high emotional exhaustion, high depersonalization and low professional fulfillment. The instrument was developed in 1981 and the Brazilian version was translated in 2001, presenting a Cronbach’s alpha coefficient from 0.65 to 0.94^15^. The Maslach Burnout Inventory is the instrument most frequently adopted worldwide because it was the first one developed and is considered the gold standard for the assessment of Burnout Syndrome in various professions, including nursing^16^ ^-^ ^17^. For these reasons, this theoretical methodological framework was chosen for this study.

The DCSQ was developed in 1988 and translated and validated for Brazil in 2004^18^, presenting appropriate psychometric properties and a Cronbach’s alpha between 0.63 and 0.86. This questionnaire has 17 items with a four-point Likert scale assessing three dimensions: psychological demands (five items); work control (six items); and social support received at work (six items).

Data were analyzed using the Statistical Package for the Social Sciences (SPSS), version 20.0. There were no missing data. The Cronbach’s alpha coefficient was used to assess the internal consistency of the MBI-HSS and DCSQ, considering α>0.70 as appropriate. Data were described using absolute and relative frequencies. Pearson’s chi-square test and Fisher’s exact test were used to determine differences between the participants of each shift.

The dependent variables were the dimensions of Burnout Syndrome: emotional exhaustion, depersonalization, and professional fulfillment, which were dichotomized into high and low, considering the median as the cutoff point^19^. Univariate binary logistic regressions were performed according to work shift to investigate the relationships among dependent and independent variables (sociodemographic and occupational characteristics, life habits and the DCSQ dimensions). 

The independent variables that presented p<0.20 (recommended to identify potential associated factors) were organized in decreasing order according to significance and likelihood ratio. Multiple models using logistic regression according to the stepwise forward method were developed. That is, the models began with the independent variable having the highest significance and then each of the remaining variables were added according to a predetermined order. Variables with statistical significance (p<0.05) and adjustment variables remained in the model. All analyzed data were adjusted by the variables: number of jobs, according to statistical criterion of adjusting values of β, in at least10%; and sex, because the literature suggests it is an aspect to be controlled for^7^ ^,^ ^20^. The results were expressed according to odds ratio (OR) with confidence intervals. Goodness-of-fit of the final model was verified using the Hosmer-Lemeshow test, in which the greater the p-value, the better the fit.

This study was approved by the Institutional Review Board (CAAE No. 57591816.3.0000.5231) and complied with ethical recommendations according to National and International norms, including permission for using the MBI-HSS with 502 individuals. The study was financed by the researchers themselves.

## Results

The response rate was 98.43%, with 502 of the 510 eligible workers participating in the study: 271(53.98%) worked on the day shift and 231(46.02%) worked on the night shift; 488(97.21%) worked 42 hours/week. Most of the participants who did not have children were sedentary, and were satisfied with their sleep worked on the day shift. The professionals older than 41 years old, male, with higher salaries, who had worked for a longer period in the institution, and who were satisfied with their leisure worked on the night shift (Table 1).

**Table 1:** Sociodemographic and occupational characteristics and life habits of nursing workers, according to work shift (n=502). Londrina, PR, Brazil, 2016

Variable	Work shift		*p-value*
	Day n(%)	Night n(%)	
Age group			
20-40 years old	224(57.00)	169(43.00)	<0.01
≥41 years old	47(43.12)	62(56.88)	
Sex			
Male	20(41.67)	28(58.33)	0.05
Female	251(55.29)	203(44.71)	
Marital status			
Single	122(51.26)	116(48.74)	0.14
Married/stable union	149(56.44)	115(43.56)	
Children			
No	118(59.90)	79(40.10)	0.02
Yes	153(50.16)	152(49.84)	
Religion			
No	18(47.37)	20(52.63)	0.25
Yes	253(54.53)	211(45.47)	
Physical activity			
Sedentary	188(56.97)	142(43.03)	0.04
Physically active^*^	83(48.29)	89(51.74)	
Smoking			
No	260(54.74)	215(45.26)	0.11
Yes	11(40.74)	16(59.26)	
Satisfied with sleep			
No	124(46.79)	141(53.21)	<0.01
Yes	147(62.03)	90(37.97)	
Satisfied with leisure			
No	213(59.66)	144(40.34)	<0.01
Yes	58(40.00)	87(60.00)	
Education			
High School	164(55.03)	134(44.97)	0.57
Higher education	107(52.45)	97(47.55)	
Profession			
Nurse	100(51.81)	93(48.19)	0.25
Nursing technician/aid	171(55.34)	138(44.66)	
Time working in the institution			
1-2 years	167(61.62)	104(38.38)	<0.01
≥3 years	104(45.02)	127(54.98)	
Other job			
No	228(54.81)	188(45.19)	0.24
Yes	43(50.00)	43(50.00)	
Monthly wage^†^			
1-2 times the minimum wage	203(56.70)	155(43.30)	0.03
3-5 times the minimum wage	68(47.22)	76(52.78)	
Satisfied with financial resources			
No	239(54.82)	197(45.18)	0.20
Yes	32(48.48)	34(51.52)	

* Level of physical activity needed to obtain health benefits (≥3x and/or ≥150 minutes/week)^(21-23)^; ^†^Minimum wage in 2016: R$880.00

The levels of the Burnout dimensions and those of the DCSQ significantly differed between work shifts, with the exception of depersonalization and work control. Thus, the levels of Burnout and DCSQ were at the highest among nursing workers from the day shift (Table 2).

**Table 2:** Reliability and comparison between the dimensions of Burnout Syndrome and the Demand-Control-Support Questionnaire among nursing workers, according to work shift (n=502). Londrina, PR, Brazil, 2016

Dimensions	Cronbach’s alpha	Work shift		*p-value*
		Day n(%)	Night n(%)	
Emotional exhaustion	0.90			
Low		132(51.16)	126(48.84)	0.04
High		139(56.97)	105(43.03)	
Depersonalization	0.71			
Low		156(54.17)	132(45.83)	0.92
High		115(53.74)	99(46.26)	
Professional realization	0.78			
Low		165(58.72)	116(41.28)	0.02
High		106(47.96)	115(52.04)	
Demand	0.76			
Low		179(51.73)	167(48.27)	0.04
High		92(58.97)	64(41.03)	
Control	0.70			
Low		145(53.70)	125(46.30)	0.89
High		126(54.31)	106(45.69)	
Social support	0.76			
Low		177(58.61)	125(41.39)	0.01
High		94(47.00)	106(53.00)	

The multiple model indicates that dissatisfaction with sleep, high demand and low control over work significantly increased the likelihood of those working the day shift to experience emotional exhaustion. High depersonalization was associated with being a nurse, sedentary and dissatisfied with sleep. Sedentariness was significantly associated with low professional fulfillment. In turn, satisfaction with financial resources decreased the likelihood of high depersonalization and low professional fulfillment (Table 3).

**Table 3:** Multiple models for the three dimensions of Burnout Syndrome among nursing professionals working on the day shift (n=271). Londrina, PR, Brazil, 2016

Variables	*Odds ratioraw* (confident interval 95%)	*p-value*	*Odds ratioadjusted* (confident interval 95%)	*p-value*
Emotional exhaustion^*†^				
Dissatisfied with sleep	2.14(1.26-3.63)	<0.01	2.20(1.31-3.72)	<0.01
Demand (high)	2.48(1.42-4.33)	<0.01	2.50(1.44-4.35)	<0.01
Control (low)	2.43(1.44-4.13)	<0.01	2.43(1.44-4.11)	<0.01
Social support (low)	1.78(1.02-3.11)	<0.01	1.87(1.08-3.25)	0.03
Depersonalization^*†^				
Social support (low)	3.73(1.53-4.86)	<0.01	2.65(1.48-4.75)	<0.01
Nurse	1.96(1.07-3.89)	0.04	1.95(1.08-3.88)	0.04
Sedentariness	1.74(1.09-3.15)	0.04	1.80(1.00-3.25)	0.05
Satisfied with financial resources	0.53(0.22-0.89)	0.01	0.64(0.46-0.88)	<0.01
Dissatisfied with sleep	1.76(1.04-3.02)	0.03	1.88(1.11-3.17)	0.01
Low Professional Realization^*†^				
Social support (low)	2.30(1.35-3.93)	<0.01	2.41(1.42-4.09)	<0.01
Sedentariness	1.90(1.09-3.34)	<0.01	2.10(1.22-3.62)	<0.01
Satisfied with financial resources	0.38(0.17-0.83)	0.01	0.38(0.17-0.82)	0.01

* Adjustment variables: number of jobs, sex, Hosmer-Lemeshow test of adjusted models: 0.72, 0.93 and 0.79, respectively.

Table 4 shows that those working on the night shift who were dissatisfied with their sleep were more likely to experience emotional exhaustion, while those without children were less likely. Professionals working for three years or more in the institution were more likely to experience high levels of depersonalization. Those who reported satisfaction with leisure and had a religion were less likely to experience a high level of depersonalization and a low level of professional fulfillment, respectively. Nursing technicians and aids were more likely to experience low professional fulfillment. 

**Table 4:** Multiple models for the three dimensions of Burnout Syndrome among the nursing workers from the night shift (n=231). Londrina, PR, Brazil, 2016

Variables	*Odds ratioraw* (confidence interval 95%)	*p-value*	*Odds ratioadjusted* (confidence interval 95%)	*p-value*
Emotional exhaustion^*†^				
Dissatisfaction with sleep	2.58(1.44-4.63)	<0.01	2.35(1.30-4.25)	<0.01
Social support (low)	2.30(1.27-4.17)	<0.01	2.62(1.47-4.67)	<0.01
No children	0.33(0.18-0.62)	<0.01	0.33(0.18-0.61)	<0.01
Depersonalization^*†^				
Time working in the facility	4.56(2.43-8.57)	<0.01	4.80(2.52-9.16)	<0.01
Social support (low)	3.44(1.85-6.38)	<0.01	3.45(1.86-6.50)	<0.01
Satisfied with leisure	2.79(1.47-5.29)	<0.01	3.02(1.56-5.84)	<0.01
Low professional realization^*†^				
Social support (low)	4.04(2.29-7.14)	<0.01	4.09(2.33-7.20)	<0.01
Having a religion	0.34(0.12-0.96)	0.04	0.33(0.12-0.93)	0.04
Nursing technicians and aids	2.18(1.21-3.91)	<0.01	2.17(1.21-3.89)	<0.01

* Adjustment variables: number of jobs; sex; ^†^Hosmer-Lemeshow test of the adjusted models: 0.71, 0.32 and 0.99, respectively.

Low social support was associated with all the dimensions of Burnout Syndrome, regardless of work shift.

## Discussion

Characterization per work shift shows that those working on the night shift were mostly older male professionals, with higher salaries, who have worked for longer periods in the facility. It is possible this is a way to benefit older workers who have worked longer in the facility, because the night shift has fewer demands compared to the day shift and higher salaries^4^. Even though there is some indication that recently graduated young and single workers with fewer years of experience^24^ work on the night shift, recent studies report results similar to this study^2^ ^,^ ^25^. 

The levels of emotional exhaustion and low professional fulfillment were significantly higher among nursing professionals on the day shift, which may be related to the fact that a larger number of young women in stable relationships work in this period, which are risk factors for Burnout Syndrome^6^ ^,^ ^26^ ^-^ ^27^. Additionally, the work process is more intense during the day, as there is more strenuous demand due to the greater number of care and nursing procedures, while interpersonal relationships are more frequently established with the multidisciplinary team, due to medical consultations and consultations provided by other health workers, which increase occupational stress and the development of Burnout Syndrome^6^ ^,^ ^28^. Such a fact was corroborated by the multiple model of high emotional exhaustion evidenced for this study’s participants.

In regard to factors associated with high emotional exhaustion, other studies also provide evidence that workload and emotional demands were positively associated, while autonomy in performing tasks (work control) and social support was negatively related to the syndrome^29^ ^-^ ^30^. 

High levels of depersonalization were 95.10% (OR:1.95) greater among nurses in comparison to nursing technicians and aids. Even though they have greater autonomy and control over their work tasks, they are in greater demand and work at a more intense pace, which predisposes them to mental diseases^31^ and, consequently, disengagement from work.

Most nursing professionals working the day shift were sedentary, a factor associated with high levels of depersonalization and low professional fulfillment. Longitudinal studies conducted with health workers show that physical exercise benefits mental health. These studies also report that the greater the intensity of physical exercises, the lower the levels of burnout, anxiety and depression, because exercises improve mental energy and decreases work fatigue by releasing neurotransmitters, such as serotonin, which produce a feeling of wellbeing. Additionally, it is a protective factor for various chronic conditions, such as cardiovascular diseases^32^ ^-^ ^33^. 

Given the physical effort required and lack of time due to the numerous daily activities, which are common motivations for sedentariness, changing behavior is not an easy task, but is one that should be attempted^33^. Therefore, managers should encourage workers to perform moderate to intense aerobic exercise for at least 150 minutes a week, with a minimum of 30 uninterrupted minutes, considering the benefits it provides to health, wellbeing and quality of life^21^ ^-^ ^23^.

The nursing workers satisfied with their salaries were more involved with their work and considered themselves to be efficient professionals. This finding is possibly linked to professional acknowledgment because nursing is a profession with low salaries and is not remunerated in accordance with the qualification required and functions that the professionals perform. Therefore, earning a salary that is higher than those earned by co-workers leads individuals to be more committed to their jobs and feel more competent^34^. 

Dissatisfaction with sleep patterns was associated with emotional exhaustion among workers of both shifts, as well as a high level of depersonalization among those working the day shift. Studies report that occupational stress and Burnout Syndrome are related to worse quality of sleep among those under shift work schemes. Additionally, various sleep disorders, such as insomnia, sleeping difficulty, early awakenings, non-restorative sleep, somnolence, short periods of sleep (fewer than 6 hours) and sleep deprivation were reported by individuals with high levels of Burnout Syndrome^12^ ^,^ ^35^. 

Nursing workers believe that the night shift is a positive aspect in their lives, considering they manage to reconcile their schedule and plan their private and professional lives. They also have some perceived advantages, such as working hours with less supervision, fewer demands, and for the most part, better salaries because of the additional night premium, and greater proximity with the members of their team. It is a fact, however, that the night shift causes disorder in workers’ biological rhythms, leading to diseases of a mental and physical nature^12^ ^,^ ^36^. 

For those working on the night shift, not having children, being satisfied with leisure and having a religion, were protective factors against high levels of exhaustion and depersonalization, and low professional fulfillment, respectively. Priorities people establish lead them to relegate leisure, rest, and family life. Leisure, however, contributes to various aspects of life, especially, biopsychosocial health, in addition to preventing and treating Burnout Syndrome^37^. Having a religious belief strengthens people when coping with stress and work problems, and often attenuates the negative impact of these on mental health^38^.

Among those working the night shift, having worked for longer periods in the institution was associated with less involvement with work. There is no consensus in the literature in regard to this aspect. Some studies report that experienced nurses are more committed to their work, and more resilient when coping with unpredictable and stressful situations, manifesting lower levels of depersonalization^39^ ^-^ ^41^. Other studies, however, show workers become more insensitive and detached as a way to protect themselves against fatigue caused by compassion and avoiding distress; that is, they aim to protect their mental health^42^ ^-^ ^43^. 

Such a divergence may be explained by individual coping strategies. For some, working with people who are facing distress is a motive for feeling distressed themselves, so they attempt to detach from the source of distress as a self-defense strategy. Other workers, however, have a sense of satisfaction when they help those in need, increasing their engagement with work^44^. 

Nursing technicians and aids were more likely to experience low professional fulfillment. Despite the importance of their work, these professionals have less autonomy in comparison to the remaining members of the health staff and are less recognized and appreciated, which may lead to a sense of uselessness and incompetence^45^. 

Low social support was associated with all the dimensions of Burnout Syndrome, regardless of work shift, a result that is similar to that reported by a study conducted in Greece^46^. Social support provided by supervisors and co-workers is essential to avoid Burnout Syndrome among nursing workers because, as these professionals experience the same situations, they can exchange impressions and promote a friendlier environment of mutual support^47^ ^-^ ^48^. 

In this sense, workplace incivility, manifested through behaviors that violate social norms of courteous and respectful conduct, has been indicated as one of the main predictors of Burnout Syndrome^49^ ^-^ ^50^ and this is why working relationships in the healthcare context are important protective factors for this syndrome.

Given the multiple consequences of Burnout Syndrome, such as adverse events experienced during care delivery, decreased quality of care delivery, decreased wellbeing, absenteeism, and increased rates of presenteeism and turnover, managers and the workers themselves need to be sensitized to the problem and make an effort to promote healthier workplaces^9^.

This study’s limitations include its cross-sectional design, which does not allow causal relationships to be established or the “effect of a healthy worker” because it was not possible to determine whether there were individuals on sick leave caused by Burnout. All the information collected was self-reported and the responses may be affected by the respondents’ interests and attitudes. As the authors of the MBI do not recommend establishing cutoff points in the dimensions nor a global score, we could not establish how prevalent the syndrome was. Another limitation involves the convenience sampling of a philanthropic hospital, which impedes the generalization of results.

Nevertheless, this study contributes to knowledge in the field, as it shows that the levels of Burnout Syndrome were greater among nursing professionals working the day shift in comparison to those on the night shift; associated factors were also different. Thus, this study can support future studies addressing interventions so that workers and managers work together to devise strategies that take into account associated factors to avoid or decrease levels of Burnout Syndrome and, consequently, maximize quality of life in the work environment, improving the care delivered to patients and families. 

## Conclusion

High levels of Burnout Syndrome were found among the nursing professionals working on the day shift. Psychosocial factors and those from the work context were associated with the dimensions of Burnout Syndrome in both shifts: in the day shift the associated factors included greater demand, low control, low social support, dissatisfaction with sleep and with financial resources, being a nurse, and sedentariness. For the night shift, the associated factors were: low social support; dissatisfaction with sleep and leisure; having children; not having a religion; fewer years working in the institution; and being a nursing technician or aid.

The differences in the levels of Burnout Syndrome and associated factors between shifts suggest that preventive strategies and measures to decrease the syndrome should be individualized according to the shift worked, possibly focusing on physical exercise but mainly on promoting social support at work.
